# MARS: a motif-based autoregressive model for retrosynthesis prediction

**DOI:** 10.1093/bioinformatics/btae115

**Published:** 2024-02-29

**Authors:** Jiahan Liu, Chaochao Yan, Yang Yu, Chan Lu, Junzhou Huang, Le Ou-Yang, Peilin Zhao

**Affiliations:** College of Electronic and Information Engineering, Shenzhen University, Shenzhen 518060, Guangdong, China; Guangdong Key Laboratory of Intelligent Information Processing, Shenzhen University, Shenzhen 518060, Guangdong, China; Shenzhen Key Laboratory of Media Security and Guangdong Laboratory of Artificial Intelligence and Digital Economy (SZ), Shenzhen University, Shenzhen 518060, Guangdong, China; Computer Science and Engineering Department, University of Texas at Artlington, Arlington 76019, TX, United States; Tencent AI Lab, Shenzhen 518057, Guangdong, China; Tencent AI Lab, Shenzhen 518057, Guangdong, China; Computer Science and Engineering Department, University of Texas at Artlington, Arlington 76019, TX, United States; College of Electronic and Information Engineering, Shenzhen University, Shenzhen 518060, Guangdong, China; Guangdong Key Laboratory of Intelligent Information Processing, Shenzhen University, Shenzhen 518060, Guangdong, China; Shenzhen Key Laboratory of Media Security and Guangdong Laboratory of Artificial Intelligence and Digital Economy (SZ), Shenzhen University, Shenzhen 518060, Guangdong, China; Tencent AI Lab, Shenzhen 518057, Guangdong, China

## Abstract

**Motivation:**

Retrosynthesis is a critical task in drug discovery, aimed at finding a viable pathway for synthesizing a given target molecule. Many existing approaches frame this task as a graph-generating problem. Specifically, these methods first identify the reaction center, and break a targeted molecule accordingly to generate the synthons. Reactants are generated by either adding atoms sequentially to synthon graphs or by directly adding appropriate leaving groups. However, both of these strategies have limitations. Adding atoms results in a long prediction sequence that increases the complexity of generation, while adding leaving groups only considers those in the training set, which leads to poor generalization.

**Results:**

In this paper, we propose a novel end-to-end graph generation model for retrosynthesis prediction, which sequentially identifies the reaction center, generates the synthons, and adds motifs to the synthons to generate reactants. Given that chemically meaningful motifs fall between the size of atoms and leaving groups, our model achieves lower prediction complexity than adding atoms and demonstrates superior performance than adding leaving groups. We evaluate our proposed model on a benchmark dataset and show that it significantly outperforms previous state-of-the-art models. Furthermore, we conduct ablation studies to investigate the contribution of each component of our proposed model to the overall performance on benchmark datasets. Experiment results demonstrate the effectiveness of our model in predicting retrosynthesis pathways and suggest its potential as a valuable tool in drug discovery.

**Availability and implementation:**

All code and data are available at https://github.com/szu-ljh2020/MARS.

## 1 Introduction

Retrosynthesis prediction is a fundamental problem in the field of organic chemistry, which plays a crucial role in the planning of chemical synthesis and drug discovery. E. J. Corey first proposed the concept of retrosynthesis, which triggered extensive research in this area. The aim of retrosynthesis prediction is to identify physically feasible reactants that can be used to synthesize target molecules, given the knowledge of their chemical structure. However, the complexity of the chemical search space makes this task highly challenging. There are approximately $10^{7}$ reactions and molecules in the published synthetic–organic knowledge (Gothard *et al.* 2012), leading to an enormous number of possible combinations that need to be considered. Traditionally, chemists relied on their experience and knowledge to derive potential reactants, which was highly inefficient and limited in scope. For example, the complete synthetic route of vitamin B12 required the collaboration of hundreds of chemists led by Robert Woodward (Woodward 1973) and took 11 years to complete. To overcome these limitations, chemists have turned to computer-aided synthesis planning (CASP) tools (Han *et al.* 2022, Liu *et al.* 2023) to design synthetic pathways. Several rule-based systems (Kayala and Baldi 2012, Marcou *et al.* 2015) have been developed and achieve excellent results for specific reaction types, but they suffer from high complexities and have limited generalization ability on reactions outside the template library.

With the development of deep learning (Otter *et al.* 2020, Wu *et al.* 2020, Meng *et al.* 2023a), deep models have spawned a series of promising proposals, greatly increasing the efficiency of synthetic route design (Meng *et al.* 2023b). These models can be categorized into two types: template-based (Coley *et al.* 2017, Segler and Waller 2017, Dai *et al.* 2019, Yan *et al.* 2022) and template-free (Yan *et al.* 2020, Shi *et al.* 2020a, Mao *et al.* 2021, Sun *et al.* 2021). Template-based models rely on templates that are either manually extracted by experienced chemists or automatically extracted from large-scale data (Coley *et al.* 2019). The core task of these methods is to match the product and the reactants to the appropriate template, which reflects the reaction center of the target molecule in a particular type of reaction. While template-based methods offer high interpretability and can overcome the issues that traditional rule-based systems give conflicting results with functional groups (Segler and Waller 2017), they are limited by the costly subgraph matching process (Liu *et al.* 2017) and poor generalization capabilities (Thakkar *et al.* 2020).

Template-free methods can be generally divided into sequence-based and graph-based methods. Sequence-based methods treat retrosynthesis prediction as a machine translation task. These methods use an encoder–decoder model, such as LSTM (Liu *et al.* 2017) and Transformer (Karpov *et al.* 2019, Zheng *et al.* 2019) to translate Simplified Molecular Input Line Entry System (SMILES) sequences of target molecules into reactants SMILES sequences without atom-mapping and subgraph matching. Although sequence-based methods can implicitly learn reaction rules and easily scale to larger datasets, they ignore the rich topological information presented in molecular graphs and are prone to generating invalid reactant molecules. Recently, many graph-based models for retrosynthesis have gained popularity with the development of graph neural networks (GNNs). These methods typically follow a similar paradigm, consisting of reaction center identification and synthon completion. G2Gs (Shi *et al.* 2020a), RetroXpert (Yan *et al.* 2020), and GraphRetro (Somnath *et al.* 2021) all use a two-stage framework to formulate the above two subtasks. However, due to the different optimization objectives of the two separate models, the two-stage approach may not achieve optimal results and can suffer from poor generalization. Additionally, GraphRetro’s use of leaving groups to complete synthons can result in unbalanced training samples. MEGAN (Sacha *et al.* 2021) is an end-to-end model that completes synthons with tiny units like single atoms and benzene, and G2GT (Lin *et al.* 2023) employs a graph-to-graph framework to generate a graph sequence in an autoregressive way, but the lengthy prediction process makes the reactant generation challenging.

**Figure 1.**
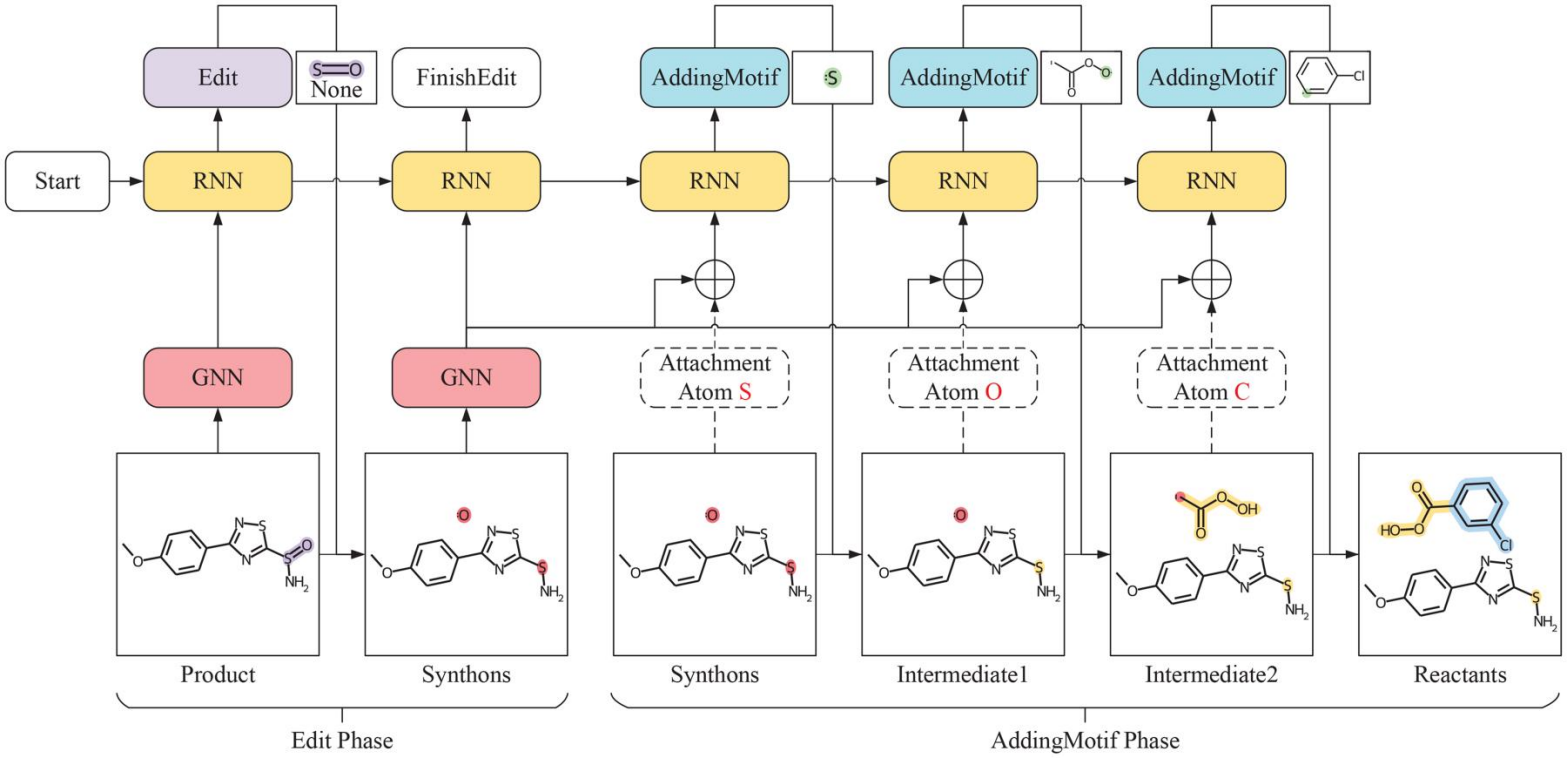
Reactants generation procedure of the proposed MARS. *Edit* and *AddingMotif* indicate graph transformation actions, where the *Edit* phase describes bond and atom changes from product to synthons and plays the role of reaction center identification while *AddingMotif* phase conducts synthon completion by adding proper motifs to synthons. Input molecular graphs are encoded by the GNN, and the RNN predicts graph transformation operations sequentially. In *Edit* phase, the RNN predicts a sequence of *Edit* operations until the *FinishEdit* which indicates the end of *Edit* phase as well as the start of *AddingMotif* phase. In *AddingMotif* phase, the RNN adds motifs sequentially until no attachment atoms remain. In the above example, the first *Edit* operation applies to the S = O bond, and the new bond type is None which indicates removing the bond. For the *AddingMotif* operation, the *interface-atom* in the motif and the *attachment atom* in the synthon/intermediate represent the same atom and are merged into a single atom when attaching the motif to the synthon/intermediate.

In this work, we propose a novel **M**otif-based **A**utoregressive model for **R**etro**S**ynthesis prediction (**MARS**), which jointly identifies reaction center and completes synthons in an end-to-end graph generation framework. The workflow of the entire model is shown in Fig. 1. For reaction center identification, MARS automatically predicts which bonds in a product need to be edited, without simply ignoring samples with multiple reaction centers or introducing additional tasks to predict the number of reaction centers. For synthon completion, we employ a predefined motif vocabulary from training reactions, instead of using a single atom or ring. Motifs are fine-grained components that enjoy lower redundancy, more balanced data distribution, and more generative flexibility than leaving groups, as proposed by GraphRetro (Somnath *et al.* 2021). We describe each step from product to reactants through carefully designed graph editing actions represented as a complete transformation path. Then, we adapt a recurrent neural network (RNN) model to learn to generate a transformation path in an autoregressive manner. Our main contributions to this work are summarized as:

- We integrate the two subtasks of reaction center identification and synthon completion into a unified framework, and adapt an encoder–decoder architecture for retrosynthesis prediction to train the model in an end-to-end manner.
- We extract a chemically meaningful motif vocabulary from training reactions without additional chemical knowledge, providing enhanced generative flexibility and significantly boosting overall performance.
- We provide a complete transformation path for each step from product to reactants, which allows for more understandable predictions.
- Experiments on the benchmark dataset show that our model could achieve state-of-the-art retrosynthesis performance with a Top-1 accuracy of 54.6% and 66.2% when w/o and w/reaction type, respectively.

## 2 Data processing

In this section, we will outline the process of constructing a transformation path from a product molecule to reactant molecules for autoregressive prediction. This involves key steps such as crafting the Edit sequence that characterizes the reaction center, extracting motifs, and building junction trees.

### 2.1 Notations

In this work, molecules are represented as graph G=(V,E) with *n* atoms and *m* bonds, where V is the set of atoms (nodes) and E is the set of bonds (edges). Each atom *u* is associated with a 45-dimensional feature vector $x_{u}$ that encodes information such as atom type, degree, chiral properties, and the count of hydrogen atoms, among others. When the reaction type is given, a 10-dimensional one-hot feature indicating the reaction type is added to the features of each atom. Each bond (u,v) is characterized by a 12-dimensional feature vector $x_{uv}$ that captures attributes like bond type, stereochemistry, aromaticity, and more. To compute these features, we employ the RDKit package (https://www.rdkit.org/). For ease of reference, we assign a unique index *i* to each bond and atom. Bond indices correspond to those assigned by RDKit, while atom indices are obtained by adding *m* to the indices assigned by RDKit. Furthermore, every bond is associated with a 4-dimensional one-hot vector $r_{b}$ representing its bond type, including none, single, double, and triple bonds, respectively. Additionally, both bonds and atoms are labeled with $s_{i}\in \{0,1\}$ indicating whether they are part of the reaction center. All the notation and their explanations are organized in Supplementary Section B.

### 2.2 Transformation path construction

**Figure 2.**
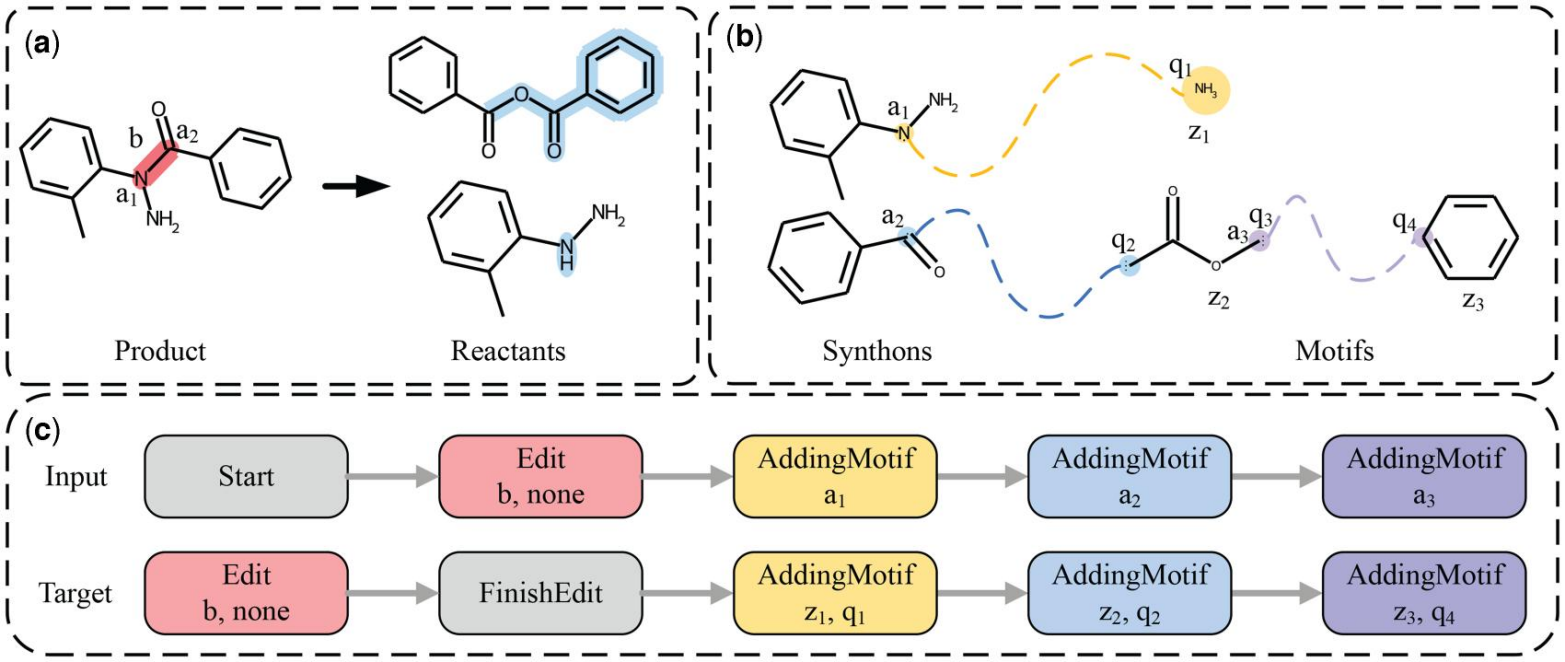
The details of transformation path construction. (**a**) The conversion process from products to reactants. The bond *b* serves as the reaction center, while the atoms $a_{1}$ and $a_{2}$ are designated as attachment atoms; (**b**) The junction tree generated using synthons and motifs $z_{1}$, $z_{2}$, and $z_{3}$. Atoms sharing the same color signify the connection between attachment atoms and interface-atoms $q_{1}$, $q_{2}$, $q_{3}$, and $q_{4}$, which are identical atoms in the reactant. The arrows symbolize parent nodes directing to the child nodes. When synthons are linked with $z_{2}$, the interface atom $q_{3}$ transforms into attachment atom $a_{3}$; (**c**) The training input and target transformation paths for RNN model, formulated in accordance with (a) and (b).

In MARS, we formulate the retrosynthesis prediction as a graph generation problem. Specifically, MARS involves predicting a sequence of graph editing actions that transform a given product into its corresponding reactants. To facilitate this, we pre-construct a transformation path for each product, consisting of two primary phases: the *Edit* phase and the *AddingMotif* phase (as shown in Fig. 1). The *Edit* phase plays the role in identifying reaction center and describing the bond and atom changes from the product to synthons. The *AddingMotif* phase, on the other hand, constructs synthon completion by adding appropriate predefined motifs to synthons. In particular, we introduce a junction tree (Jin *et al.* 2018) to represent the connection between synthons and motifs (Fig. 2b), which provides an efficient way to create *AddingMotif* sequences. To seamlessly integrate *Edit* and *AddingMotif* actions into a complete transformation path, we define four graph transformation tokens: *Start*, *Edit*, *FinishEdit*, and *AddingMotif*. Except for auxiliary actions *Start* and *FinishEdit*, each token in the transformation path comprises three parts: edit action π, edit object *o*, and edit state τ. We further elaborate the representation of the reaction center in the Edit sequence, motif extraction, and junction tree construction.

#### 2.2.1 Edit sequence construction

The *Edit* phase of MARS encompasses two types of edits: bond edit and atom edit. Bond edit involves adding or removing a bond or changing the bond type between two heavy atoms, while atom edit pertains to altering the number of hydrogen atoms or the charge of an atom. These edits are applied to the target molecule to generate synthons. Atoms at both ends of the modified bonds, as well as atoms with altered hydrogen counts or charges, are referred to as *attachment atoms*.

As shown in Fig. 2a and c, the reaction center of the target molecule can be encoded as an *Edit* sequence, where each *Edit* token consists of a tuple *(edit action, edit object, edit state)*.

#### 2.2.2 Motif extraction

Through the *Edit* phase, a product molecular graph is decomposed into a set of incomplete subgraphs called synthons. By combining suitable motifs and attachments, synthons can be reconstructed into valid reactant molecular graphs. In other words, motifs represent subgraphs of reactant molecular graphs. The details of motif extraction are summarized as follows:

- Bonds connecting the synthon in the reactants are broken to obtain a set of subgraphs. Each subgraph retains the attachment atoms connected to it on the synthon, resulting in a coarse-grained motif.
- If two connected atoms belong to separate rings, the bond between them is broken, yielding two independent motifs.
- In cases where one atom belongs to a ring, and the other has a degree greater than 1, the bond between them is broken, resulting in two independent motifs.

Finally, a motif vocabulary *Z* of size |Z|=210 is obtained from the USPTO-50K training set (Schneider *et al.* 2016). It is worth noting that motifs differ fundamentally from leaving groups used in the previous method (Somnath *et al.* 2021): (i) A motif is connected to an attachment atom, whereas a leaving group is associated with a synthon. As a synthon may contain multiple attachment atoms, and a leaving group may consist of multiple disconnected subgraphs (i.e. motifs). (ii) Motif retains the corresponding attachment atom on the synthon, referred to as the *interface-atom*. Notably, many added leaving groups consist of single hydrogen atoms, leading to an extremely unbalanced frequency of leaving groups. (iii) A large leaving group may contain multiple rings or large branched chains, which appear infrequently in the dataset. To reduce redundancy, we cut these into multiple small motifs that are common in the dataset.

#### 2.2.3 Junction tree construction

Based on chemical intuition, it is postulated that reactants can be decomposed into synthons and motifs, where synthons represent molecule fragments obtained by breaking the bonds in the product, and motifs represent subgraphs of reactants. To maintain the connection between synthons and motifs, the junction tree method (Jin *et al.* 2018) is introduced. The junction tree method represents synthons and motifs as a hierarchical tree structure, where the group of synthons is set as the root node and motifs are set as children nodes (Fig. 2b). The connected edge between two nodes indicates that they are directly linked in the reactants, denoted as *(attachment, motif, interface-atom)*. The trees are traversed using depth-first search (DFS) to preserve the linked edges between nodes, and to obtain the training input and target AddingMotif paths. The input path consists of each token containing an action *AddingMotif* and an object *attachment atom*, while for target path, the object consists of the *motif z* and the *interface-atom q*.

By combining the aforementioned Edit sequence with the AddingMotif path, as well as other auxiliary actions, the input and target transformation paths corresponding to each product can be obtained.(Fig. 2c).

## 3 MARS

In this section, we will introduce the two main modules of MARS. A graph encoder module is used to extract the graph representation embedding of the molecular graph, and an autoregressive prediction module sequentially predicts the transformation path from the product molecules to the reactant molecules.

### 3.1 Graph encoder

GNNs (Kipf and Welling 2017, Hamilton *et al.* 2017, Veličković *et al.* 2017) are a series of neural network architectures specifically designed to act on graph structure and properties, updating the representation vectors (i.e. embeddings) of nodes via a message passing mechanism. In this study, we utilize the *L*-layer graph transform network (GTN) (Shi *et al.* 2021) to capture the latent representation of molecular graphs, since it can effectively handle the heterogeneity inherent in molecular graphs.

For a given product graph *G*, the calculation of atom representations $\left\{h_{u}\in R^{D}|u\in G\right\}$ can be summarized as:

(1) $$\begin{array}{c} \begin{array}{c} h_{u}=\text{GTN}(x_{u},{\left\{x_{v}\right\}}_{v\in N(u)},{\left\{x_{uv}\right\}}_{v\in N(u)}), \end{array} \end{array}$$

where GTN(·) represents GTN. For the sake of brevity, we provide an in-depth exposition of GTN in the Supplementary Section A.

Then we proceed to update both atom and bond representations by incorporating self-loop representations for atoms and the representations of the two atoms at each end of the bond, as follows:

(2) $$\begin{array}{c} h_{uu}={\text{MLP}}_{\mathrm{bond}}(h_{u}\;||\;h_{u}), \end{array}$$

(3) $$\begin{array}{c} h_{uv}={\text{MLP}}_{\mathrm{bond}}(h_{u}\;||\;h_{v}), \end{array}$$

where ${\text{MLP}}_{*}(\cdot )$ denotes a multilayer perceptron with a Mish (Misra 2019) activation function.

For convenience, we use the notation $e_{i}\in {\left\{h_{uv}\right\}}_{v\in N(u)\cup \left\{u\right\}}$ to refer to both bond and atom representations, where *i* is the index of the bond or atom. The final graph representation $h_{G}\in R^{D}$ is defined by aggregating the whole atom representations using a readout function as follows:

(4) $$\begin{array}{c} \begin{array}{c} h_{G}=\text{Readout}(\{h_{u}|u\in G\}). \end{array} \end{array}$$

We use global attention pooling function as Readout(·), and the details of the global attention pooling function and the impact of different readout functions on the performance are discussed in Supplementary Section A. Similarly, the graph representation of synthons $h_{\mathrm{syn}}\in R^{D}$ can also be computed.

### 3.2 Autoregressive model

Inspired by prior research (Popova *et al.* 2019, Shi *et al.* 2020b), we approach retrosynthesis prediction as an autoregressive conditional molecule generation challenge. In this framework, an autoregressive model progressively constructs a new graph structure $G_{t}$ based on the partially completed graph from previous steps, ultimately arriving at the reactant graph $G_{R}$. This process can be formally described as a jointly conditional likelihood function:

(5) $$\begin{array}{c} P(G_{R}|G_{P})=\prod_{t=1}^{N}P(G_{t}|G_{0},\ldots ,G_{t-1})=\prod_{t=1}^{N}P(G_{t}|G_{<t}), \end{array}$$

where *N* is the length of generated sequence and $G_{0}$ is the given product graph $G_{P}$.

It is important to note that the intermediate graph structure $G_{t}$ is not directly generated by the model. Instead, the model generates a graph editing action π, an edit object *o* (i.e. bond, atom, or motif), and its edit state τ (e.g. new bond type or interface-atom) based on the history of graph editing actions. These actions are then applied to $G_{t-1}$ to create a new graph structure. Consequently, given the history of edited objects, edited states, and incomplete graphs, the likelihood function in Equation (5) can be redefined as:

(6) $$\begin{array}{c} P(G_{R})=\prod_{t=1}^{N}P(\pi_{t},o_{t},\tau_{t}|o_{<t},\tau_{<t},G_{<t}). \end{array}$$

We utilize a gated recurrent unit (GRU) (Chung *et al.* 2014, Li *et al.* 2016), a type of RNN), to model the likelihood function described in Equation (6). The GRU effectively captures information from the previous step, including the object, state, and incomplete graph, and translates it into a D-dimension output $u_{t}\in R^{D}$. To incorporate the global topological information of $G_{P}$ in generation process, we concatenate $h_{G}$ and $u_{t}$ for subsequent prediction. Specifically, the process can be represented as follows:

(7) $$\begin{array}{c} \begin{array}{c} u_{t}=\text{GRU}(\mathrm{inpu}t_{t}),\;\mathrm{where}\;\mathrm{inpu}t_{0}=0, \end{array} \end{array}$$

where $\mathrm{inpu}t_{t}\in R^{D}$ is the input embedding of GRU at step *t*. The hidden state of GRU is initialized by $\sigma_{G}(h_{G})$, where $\sigma_{*}(\cdot )$ is a linear layer without nonlinear activation. The resulting vector $u_{t}$ is then combined with $h_{G}$ using the concatenation operation as:

(8) $$\begin{array}{c} \psi_{t}=h_{G}\;||\;u_{t}, \end{array}$$

The generation process starts with the *Start* action, and at each step *t*, we generate graph editing action ${\hat{\pi }}_{t}$ by follow:

(9) $$\begin{array}{c} \begin{array}{c} {\hat{\pi }}_{t}=\text{softmax}({\text{MLP}}_{\mathrm{act}}(\psi_{t})). \end{array} \end{array}$$

**Edit Phase**: When the predicted action is *Edit*, the process enters the Edit phase. At step *t*, the model assigns an editing score ${\hat{s}}_{i}$ to each bond and atom, indicating the likelihood that the bond or atom is a suitable candidate for editing. The editing score is computed as follows:

(10) $$\begin{array}{cc} {\hat{s}}_{i}^{t} & =\text{sigmoid}({\text{MLP}}_{\mathrm{target}}(\psi_{t}\;||\;\sigma_{e}(e_{i}))). \end{array}$$

The atom or bond with the largest editing score is selected as the edit object, and the atom or atoms at both ends of the selected bond are designated as attachment atoms. The model then predicts the new bond type ${\hat{r}}_{b}$ for the edit object as:

(11) $$\begin{array}{cc} {\hat{r}}_{b}^{t} & =\text{softmax}({\text{MLP}}_{\mathrm{type}}(\psi_{t}\;||\;\sigma_{e}(e_{\underset{i}{\arg\max}({\hat{s}}_{i}^{t})}))). \end{array}$$

The synthon structure is then modified by applying the edit object and its new bond type. The resulting structure is embedded using GTN(·) to obtain the synthon embedding $h_{t}^{\mathrm{syn}}$. Finally, $\mathrm{inpu}t_{t}$ is updated by the synthon embedding, edit object, and its new bond type:

(12) $$\begin{array}{cc} \mathrm{inpu}t_{t+1} & =f_{\pi }({\hat{\pi }}_{t})+\sigma_{e}(e_{\underset{i}{\arg\max}({\hat{s}}_{i}^{t})})+f_{b}({\hat{r}}_{b}^{t})+h_{\mathrm{syn}}^{t}, \end{array}$$

where $f_{*}(\cdot )$ is a linear layer without activation functions, mapping entities to vectors. The model iterates this process to generate an Edit sequence that covers all reaction centers. When the model predicts the action to be a *FinishEdit*, the Edit phase ends and AddingMotif phase begins. The synthon structure is fixed and its embedding is denoted as $h_{\mathrm{syn}}$. Assume that after $N_{1}$ Edit operations, a total of $N_{2}$ attachment atoms $\left\{a_{1},\ldots ,a_{N_{2}}\right\}$ are obtained, where $a_{j}$ is the atom index in target molecular graph $G_{P}$. Then the set of attachment atoms is sorted, and $\mathrm{inpu}t_{t+1}$ is updated as:

(13) $$\begin{array}{c} \mathrm{inpu}t_{t+1}=f_{\pi }({\hat{\pi }}_{t})+\sigma_{\mathrm{att}}(e_{m+a_{t}})+h_{\mathrm{syn}}. \end{array}$$

**AddingMotif Phase**: In this phase, the model traverses all attachment atoms $\left\{a_{1},\ldots ,a_{N_{2}}\right\}$ sequentially, assigning an appropriate motif to each attachment. Motif prediction is treated as a multi-classification task on the motif vocabulary *Z*. Once the predicted motif ${\hat{z}}_{t}$ is obtained, the model determines which interface-atom on the motif corresponds to the attachment atom $a_{t}$. To achieve this, the model predicts both the motif ${\hat{z}}_{t}$ and interface-atom index ${\hat{q}}_{t}$ as follows:

(14) $$\begin{array}{cc} {\hat{z}}_{t} & =\text{softmax}({\text{MLP}}_{\mathrm{motif}}(\psi_{t})), \end{array}$$

(15) $$\begin{array}{c} {\hat{q}}_{t}=\text{softmax}({\text{MLP}}_{\mathrm{interface}}(\psi_{t}\;||\;f_{z}({\hat{z}}_{t}))). \end{array}$$

If the predicted motif ${\hat{z}}_{t}$ contains only one interface-atom, the input representation $\mathrm{inpu}t_{t+1}$ is computed as Equation (13). However, if ${\hat{z}}_{t}$ contains multiple interface-atoms, and ${\hat{q}}_{t}$ is the index, $\mathrm{inpu}t_{t+1}$ is updated as follows:

(16) $$\begin{array}{c} \begin{array}{c} \mathrm{inpu}t_{t+1}=f_{\pi }({\hat{\pi }}_{t})+f_{z}({\hat{z}}_{t})+f_{\mathrm{interface}}({\hat{q}}_{t})+h_{\mathrm{syn}}. \end{array} \end{array}$$

It is important to note that there is no need for an action indicating the end of the process. The generation process continues until all attachments on the synthons and added motifs have been traversed. Finally, the model produces a transformation path, which is applied to the product to obtain reactants.

### 3.3 Training and inference

MARS is trained to predict target transformation paths using cross-entropy loss $L_{c}$ for predicting new types, motifs, and interface-atom indexes, and binary cross-entropy loss $L_{b}$ for predicting reaction centers. Teacher-forcing (Williams and Zipser 1989) is used to facilitate training.

During inference, we employ the beam search algorithm (Tillmann and Ney 2003) with hyperparameter *k* to rank predictions. The Top-*k* best results are selected at each step based on the log-likelihood score. Importantly, atom-mapping is unnecessary in the inference phase.

The pseudocode and implementation details of MARS are described in Supplementary Section A.

## 4 Results

### 4.1 Experiment setup

#### 4.1.1 Data

We evaluate the effectiveness of MARS on a widely used benchmark dataset called USPTO-50K (Schneider *et al.* 2016). This dataset includes a collection of 50K reactions from the US patent literature, which are categorized into 10 different classes. We follow the same training/validation/testing splits in an 8:1:1 ratio, as previously established in (Coley *et al.* 2017, Dai *et al.* 2019). Notably, the USPTO dataset has been reported to contain a shortcut in 75% of the product molecules, where the atom of atom-mapping “1” is part of the reaction center. To address this issue, we eliminate these shortcuts by canonicalizing product SMILES and reassigning atom-mapping to reactant atoms.

#### 4.1.2 Evaluation

We use the standard Top-*k* accuracy metric to evaluate our model’s performance. This metric measures the percentage of correct ground truth reactants found within the Top-*k* suggestions generated by our model. To calculate accuracy, we compare the predicted reactants to the ground truth reactants, both represented in canonical SMILES format.

#### 4.1.3 Baseline

We evaluated MARS against three template-based and seven template-free methods. For template-based models, we considered RetroSim (Coley *et al.* 2017), NeuralSym (Segler and Waller 2017), and GLN (Dai *et al.* 2019). For template-free models, our evaluation encompassed five sequence-based models: SCROP (Zheng *et al.* 2019), RetroPrime (Wang *et al.* 2021), Retroformer (Wan *et al.* 2022), DualTF (Sun *et al.* 2021), and Chemformer (Irwin *et al.* 2022). Additionally, we assessed five graph-based models: G2Gs (Shi *et al.* 2020a), RetroXpert (Yan *et al.* 2020), GraphRetro (Somnath *et al.* 2021), MEGAN (Sacha *et al.* 2021), and G2GT (Lin *et al.* 2023). Descriptions of baselines are provided in Supplementary Section B.

All results are derived from their original reports, except for NeuralSym reported by GLN, and corrected results reported by RetroXpert on their website (https://github.com/uta-smile/RetroXpert).

### 4.2 Overall performance

In Table 1, we present the Top-*k* accuracy results, with *k* ranging from {1,3,5,10}, for both unknown and known reaction types.

**Table 1.** Top-*k* accuracy for retrosynthesis prediction on USPTO-50K.

Methods		Top-*k* accuracy (%)							
		Reaction type known				Reaction type unknown			
		1	3	5	10	1	3	5	10
Template-based	RetroSim	52.9	73.8	81.2	88.1	37.3	54.7	63.3	74.1
	NeuralSym	55.3	76.0	81.4	85.1	44.4	65.3	72.4	78.9
	GLN	64.2	79.1	85.2	90.0	52.5	69.0	75.6	83.7
Sequence-based	SCROP	59.0	74.8	78.1	81.1	43.7	60.0	65.2	68.7
	RetroPrime	64.8	81.6	85.0	86.9	51.4	70.8	74.0	76.1
	Retroformer	64.0	82.5	86.7	90.2	53.2	71.1	76.6	82.1
	DualTF	65.7	81.9	84.7	85.9	53.6	70.7	74.6	77.0
	Chemformer					54.3		62.3	63.0
Graph-based	MEGAN	60.7	82.0	87.5	91.6	48.1	70.7	78.4	86.1
	G2Gs	61.0	81.3	86.0	88.7	48.9	67.6	72.5	75.5
	RetroXpert	62.1	75.8	78.5	80.9	50.4	61.1	62.3	63.4
	GraphRetro	63.9	81.5	85.2	88.1	53.7	68.3	72.2	75.5
	G2GT					54.1	69.9	74.5	77.7
	MARS	**66.2** ^a^	**85.8**	**90.2**	**92.9**	**54.6**	**76.4**	**83.3**	**88.5**

a The best results are bolded.

#### 4.2.1 Reaction type unknown

When dealing with unknown reaction types, MARS outperforms both template-based and template-free models. We have the following observations: (i) Our graph-based model consistently outperforms the sequence-based model, highlighting the ability of the graph-based approach to leverage richer information encoded in the graph for more accurate predictions. (ii) Our model achieves a Top-1 accuracy of 54.6%, surpassing MEGAN’s 48.1%. Although both are end-to-end methods, our motif-based approach proves more effective than strategies that rely on sequentially adding individual atoms or benzene rings to complete the synthons. This highlights the efficacy of our designed motifs. (iii) As *k* exceeds 3, our end-to-end model significantly outperforms the two-stage approach, achieving a Top-*k* accuracy more than 8.1% higher than GraphRetro. This suggests that the end-to-end model can explore the latent relationship between the reaction centers and the completion of synthons, instead of having two separate optimization objectives. Meanwhile, we note that although G2GT is designed as an end-to-end model, its performance gap widens compared to other graph-based end-to-end models when *k* exceeds 3. In comparison to the two-stage model, it exhibits only marginal improvement. This difference is attributed to the increased complexity associated with using all-atomic sequences for generation.

#### 4.2.2 Reaction type known

We have a similar observation when the type of reaction is known. Although template-based methods can use the knowledge of the reaction center to narrow down the template space and improve accuracy, our model still exhibits superior performance. MARS achieves Top-1 accuracy outperforming MEGAN by 5.5% and GraphRetro by 2.3%. Moreover, for larger values of *k*, our model achieves state-of-the-art Top-*k* accuracy, outperforming GraphRetro overall by more than 4.3%. These results collectively confirm the effectiveness and outstanding performance of our model.

### 4.3 Reaction type performance

**Figure 3.**
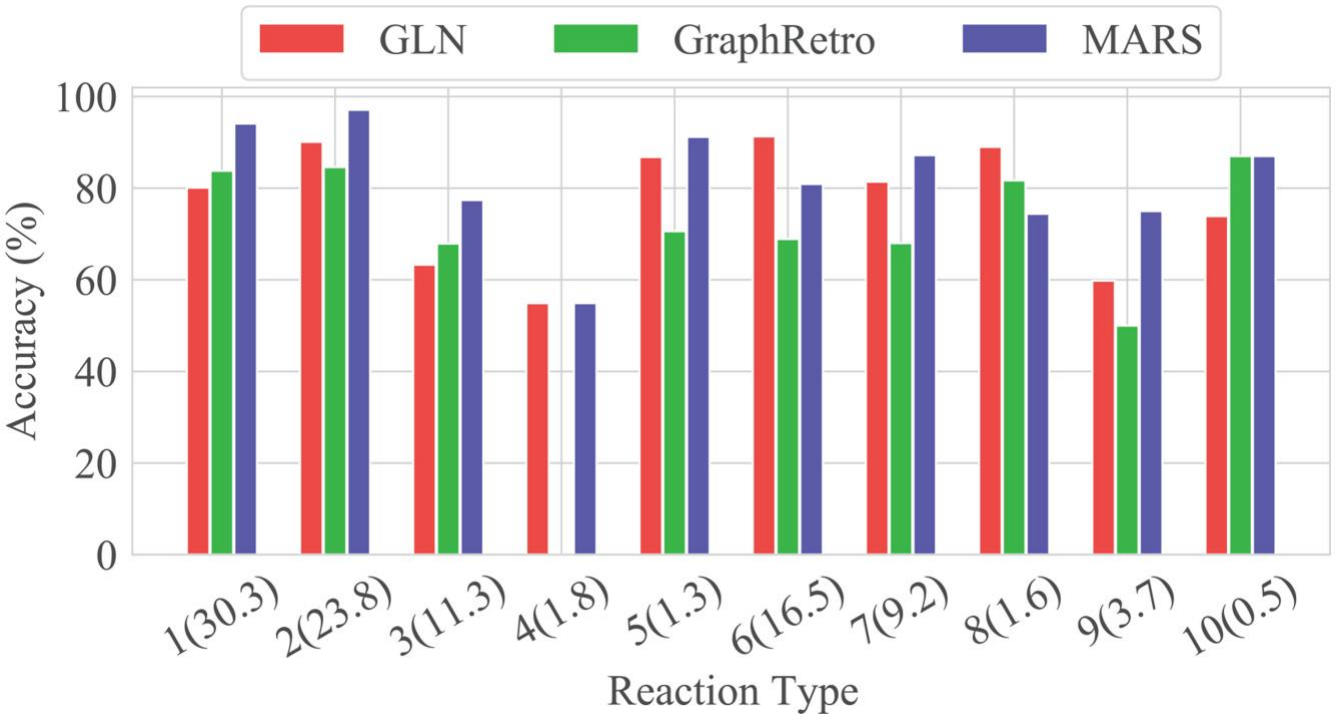
Comparison of the Top-10 accuracy across the USPTO-50K reaction types. We report the results of GLN and GraphRetro with a beam size of 10. The labels on the x-axis represent reaction types and their proportion in USPTO-50K dataset.

We further evaluate MARS’s performance for each reaction type. As shown in Fig. 3, MARS achieves competitive performance in eight categories compared with the template-based method GLN. Additionally, MARS outperforms the baseline methods on reaction types with fewer samples, such as class 5 and 9. This suggests that MARS does not suffer from overfitting even on an imbalanced dataset. Notably, reaction type 4 is heterocycle formation, which contains multiple reaction centers. GraphRetro, which only considers single reaction centers, provides inaccurate predictions for such samples. In contrast, our model reaches a Top-10 accuracy of 54.9%, on par with GLN, without requiring additional chemical knowledge.

### 4.4 Ablation study

To gain insight into the importance of synthon embedding, we conduct an ablation study by removing it from MARS. As shown in Table 2, when the synthon embedding is excluded, the Top-1 accuracy drops by 4.9% for known reaction types and 10.5% for unknown types. This demonstrates that synthon embedding is crucial for the generation process. Synthon structure information helps the model determine the *FinishEdit* action, while the absence of synthon embedding leads to the repeated prediction of edit objects in *Edit* phase.

**Table 2.** Top-*k* accuracy of synthon embedding ablation study^a^.

Method	Top-k accuracy (%)							
	1	3	5	10	1	3	5	10
	Reaction type known				Reaction type unknown			
MARS-w/o S	61.3	73.5	76.3	81.8	44.1	58.5	63.0	69.3
MARS-w/o B	64.0	84.4	89.3	92.4	51.8	74.6	81.5	86.8
MARS	**66.2**	**85.6**	**90.2**	**92.9**	**54.6**	**76.4**	**83.3**	**88.5**

a MARS-w/o S indicates MARS without synthon embedding. MARS-w/o B indicates MARS without bond feature. The best results are bolded.

In addition, we also evaluate the significance of bond features by removing them from MARS and assessing the model’s performance. The Top-*k* accuracy of MARS without bond features is presented in Table 2. Without bond features, Top-1 accuracy decreases by 2.8% for unknown reaction types and 2.2% for known types. This highlights that incorporating bond features enables MARS to learn better molecular representations, ultimately improving downstream prediction accuracy.

### 4.5 Prediction visualization

**Figure 4.**
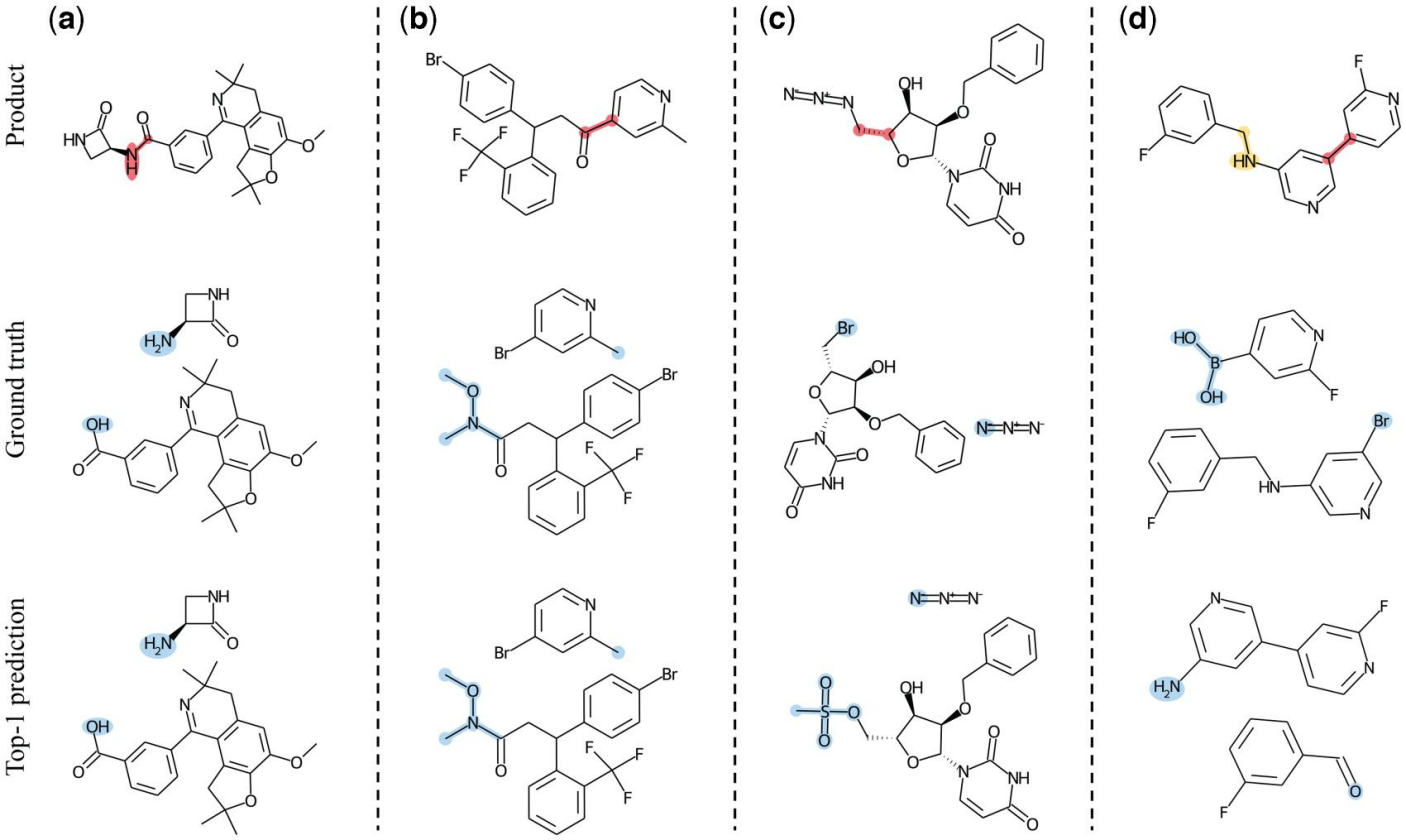
Examples of our predictions. ‘Red’ indicates the correct reaction centers while ‘yellow’ represents the error one predicted by our model, and ‘blue’ indicates the added motifs. (**a**) and (**b**) Examples of successful predictions by our model. (**c**) Correctly predicted reaction center but added the wrong motif. (**d**) Incorrectly predicted reaction center.

To provide a comprehensive understanding of the prediction performance of our model, we visually present four ground truth reactants and the Top-1 predicted reactants from the USPTO-50K test set in Fig. 4. In Fig. 4a and b, our model correctly predicts the reactants with accurate identification of the reaction centers and the addition of appropriate motifs. Remarkably, our model handles motifs of various sizes with ease, demonstrating its ability to assign the correct motifs for the synthons. Compared to methods that add atoms or benzene rings one by one, our model’s predictions demonstrate high accuracy and chemical rationality. In Fig. 4c, although our model correctly predicts the reaction center, the added motifs differ from the ground truth. However, the predicted reactants are chemically reasonable and can be more convenient to obtain in some cases. In Fig. 4d, our model predicts another disconnection site and adds corresponding motifs based on the predicted synthons. The predictions are also correct (checked by chemists), as the prediction and ground truth differ only in the disconnection order from the multi-step retrosynthesis perspective. These examples illustrate that our model can inherently learn the underlying reaction rules, providing predictions with high chemical rationality and accuracy.

## 5 Conclusion

In this work, we have introduced MARS, a graph generative model for retrosynthetic analysis. Our model benefits from the flexibility and low prediction complexity of motifs. Its end-to-end architecture empowers it to uncover latent relationships between reaction centers and motifs. Furthermore, considering that motifs correspond to fundamental chemical functional groups, treating them as elementary entities in retrosynthetic prediction is both logical and practical. These aspects collectively contribute to the remarkable performance demonstrated by our model. In the future, we plan to explore the possibility of pre-training a model to acquire a more nuanced understanding of motifs from existing chemical compounds.

## Supplementary Material

btae115_Supplementary_Data

## Data Availability

The data used in this paper is available at https://github.com/szu-ljh2020/MARS.
