# Supplementary material for: MARS: a motif-based autoregressive model for retrosynthesis prediction
Source: Bioinformatics. 2024 Feb 29;40(3):btae115. doi: 10.1093/bioinformatics/btae115 (PMC10948277; doi:10.1093/bioinformatics/btae115)
Supplement: btae115_Supplementary_Data [file btae115_supplementary_data.pdf]

A. Method Details

A.1. Graph Transformer Network

Graph Transformer Network (GTN) (Shi et al., 2020c) utilizes improved multi-head self-attention modules in vanilla transformer (Vaswani et al., 2017) to aggregate both atom and bond features. In the head c of layer l, the query $\boldsymbol{q}_{c,u}^{(l)}$, keyword $\boldsymbol{k}_{c,u}^{(l)}$, and value $\boldsymbol{v}_{c,u}^{(l)}$ vectors correspond to the atom representation vector $\boldsymbol{h}_u^{(l)}$, which are transformed by the query, keyword, and value matrices, as follows:

$$\begin{aligned} \boldsymbol{q}_{c,u}^{(l)} &= \boldsymbol{W}_{q,c}^{(l)} \boldsymbol{h}_u^{(l)}, \\ \boldsymbol{k}_{c,u}^{(l)} &= \boldsymbol{W}_{k,c}^{(l)} \boldsymbol{h}_u^{(l)}, \\ \boldsymbol{v}_{c,u}^{(l)} &= \boldsymbol{W}_{v,c}^{(l)} \boldsymbol{h}_u^{(l)}. \end{aligned} \tag{1}$$

The GTN integrates bond features $\boldsymbol{x}_{uv}$, calculates an attention score to the bond (u, v), and updates the representation of atom u by message passing scheme as follows:

$$\begin{aligned} \boldsymbol{h}_{c,uv} &= \boldsymbol{W}_{c,e} \boldsymbol{x}_{uv} + \boldsymbol{b}_{c,e}, \\ \alpha_{c,uv}^{(l)} &= \frac{\langle \boldsymbol{q}_{c,u}^{(l)}, \boldsymbol{k}_{c,v}^{(l)} + \boldsymbol{h}_{c,uv} \rangle}{\sum_{v \in \mathcal{N}(u)} \langle \boldsymbol{q}_{c,u}^{(l)}, \boldsymbol{k}_{c,v}^{(l)} + \boldsymbol{h}_{c,uv} \rangle}, \\ \hat{\boldsymbol{h}}_u^{(l+1)} &= \Big\|_{c=1}^{C} \Big[\sum_{v \in \mathcal{N}(u)} \alpha_{c,uv}^{(l)} (\boldsymbol{v}_{c,v}^{(l)} + \boldsymbol{h}_{c,uv}) \Big], \end{aligned} \tag{2}$$

where $\langle \boldsymbol{q}, \boldsymbol{k} \rangle = \exp(\frac{\boldsymbol{q}^T \boldsymbol{k}}{\sqrt{d}})$, d is the dimension of each head, $\mathcal{N}(u)$ denotes the neighbors of atom u and $\|$ is the concatenation operation. Additionally, the gated residual connection is introduced to avoid over-smoothing.

A.2. Global Attention Pooling Function

Following (Li et al., 2019), we use the global attention pooling function. Given the node representations h_v from the output layer of GNNs, the graph representation can be computed as follows:

$$h_G = \sum_{v=1}^{N} \mathrm{Softmax}(f_{gate}(h_v) f_{feat}(h_v)), \tag{3}$$

where $f_{gate}(\cdot)$ and $f_{feat}(\cdot)$ are Multilayer Perceptrons.

A.3. Objective Function

MARS is trained to predict target transformation paths using cross-entropy loss $\mathcal{L}_c$ for predicting new types, motifs, and interface-atom indexes, and binary cross-entropy loss $\mathcal{L}_b$ for predicting reaction centers. The overall loss function for MARS combines these losses across all steps in the Edit and AddingMotif sequences, which is summarized as:

$$\mathcal{L} = \sum_{t=0}^{N_1+N_2} \mathcal{L}_c(\hat{\pi}_t, \pi_t) + \sum_{t=0}^{N_1} \left[\sum_{i=0}^{n+m-1} \mathcal{L}_b(\hat{s}_i^t, s_i^t) + \mathcal{L}_c(\hat{r}_b^t, r_b^t) \right] + \sum_{t=0}^{N_2} \left[\mathcal{L}_c(\hat{z}_t, z_t) + \mathcal{L}_c(\hat{q}_t, q_t) \right], \tag{4}$$

where N_1 and N_2 denote the lengths of the Edit and AddingMotif sequences respectively.

A.4. Framework of MARS

In Algorithm 1, we present the overall workflow of the proposed method MARS.

A.5. Implementation Details

We implement MARS using PyTorch (Paszke et al., 2019) and Pytorch Geometric (Fey and Lenssen, 2019) library. The Graph Transformer consists of six eight-head self-attention modules, and employs attention pooling (Li et al., 2015) as the readout function. The GRU network comprises three layers. The embedding size D for our model is uniformly set to 512. Throughout all experiments, we conducted 100 epochs of training on USPTO-50K dataset with a batch size of 32. We employed the Adam optimizer with initial learning rate of 0.0003. To optimize learning, we employed a cosine annealing learning rate with a restart cycle of 20 epochs. Training on the USPTO-50K dataset was executed on a single NVIDIA Tesla V100 GPU, taking approximately 17 hours. During the inference phase, we used a beam size $k = 10$ to rank predictions.

B. Experimental Setting

B.1. Dataset Information

The USPTO-50K dataset comprises 50,016 reactions categorized into 10 distinct classes. The distribution of these reaction classes is presented in Table 1, illustrating the prevalence of class imbalances within the dataset, which poses a significant challenge for retrosynthesis prediction tasks.

Algorithm 1 Framework of MARS.

Require: The SMILES string of the product.
Ensure: The SMILES string of reactants.
Convert the product's SMILES to molecular graph G_P with atom features $\{\boldsymbol{x}_u\}$ and bond features $\{\boldsymbol{x}_{uv}\}$ using RDKit.
Compute graph embeddings $\{\boldsymbol{e}_i\}$ and $\boldsymbol{h}_G$ with a Graph Encoder.
Initialize π_0, $t \leftarrow 0$, hidden state of GRU $\leftarrow \sigma_G(\boldsymbol{h}_G)$, and $\boldsymbol{input}_0 \leftarrow \boldsymbol{0}$.
while π_t is not FinishEdit **do**
    $\boldsymbol{u}_t \leftarrow \mathrm{GRU}(\boldsymbol{input}_t)$.
    $\boldsymbol{\psi}_t \leftarrow \boldsymbol{h}_G \| \boldsymbol{u}_t$.
    $\hat{\pi}_t \leftarrow \mathrm{softmax}(\mathrm{MLP}_{act}(\boldsymbol{\psi}_t))$.
    $\hat{s}_i \leftarrow \mathrm{sigmoid}(\mathrm{MLP}_{target}(\boldsymbol{\psi}_t \ \| \ \sigma_e(\boldsymbol{e}_i)))$.
    $\hat{r}_b \leftarrow \mathrm{softmax}(\mathrm{MLP}_{type}(\boldsymbol{\psi}_t \ \| \ \sigma_e(\boldsymbol{e}_{\arg\max_i(\hat{\boldsymbol{s}}_i)})))$.
    $\boldsymbol{input}_{t+1} \leftarrow f_\pi(\hat{\pi}_t) + \sigma_e(\boldsymbol{e}_{\arg\max_i(\hat{\boldsymbol{s}}_i)}) + f_b(\hat{r}_b) + \boldsymbol{h}_t^{syn}$.
    $t \leftarrow t + 1$.
end while
Compute synthon graph representation $\boldsymbol{h}_{syn}$ using a graph Encoder.
Sort attachment atoms by their atom index in G_P.
$\boldsymbol{input}_t = f_\pi(\hat{\pi}_t) + \sigma_{att}(a_1) + \boldsymbol{h}_{syn}$.
for each set of attachment atoms $\{a\}$ **do**
    $\boldsymbol{u}_t \leftarrow \mathrm{GRU}(\boldsymbol{input}_t)$.
    $\boldsymbol{\psi}_t \leftarrow \boldsymbol{h}_G \| \boldsymbol{u}_t$.
    $\hat{\pi}_t \leftarrow \mathrm{softmax}(\mathrm{MLP}_{act}(\boldsymbol{\psi}_t))$.
    $\hat{z}_t \leftarrow \mathrm{softmax}(\mathrm{MLP}_{motif}(\boldsymbol{\psi}_t))$.
    $\hat{q}_t \leftarrow \mathrm{softmax}(\mathrm{MLP}_{interface}(\boldsymbol{\psi}_t \ \| \ f_z(\hat{z}_t)))$.
    if motif $\hat{z}_t$ has only one interface-atom **then**
        $\boldsymbol{input}_{t+1} \leftarrow f_\pi(\hat{\pi}_t) + \sigma_{att}(\boldsymbol{e}_{a_t}) + \boldsymbol{h}_{syn}$.
    else
        $\boldsymbol{input}_{t+1} = f_\pi(\hat{\pi}_t) + f_z(\hat{z}_t) + f_{interface}(\hat{q}_t) + \boldsymbol{h}_{syn}$.
    end if
    $t \leftarrow t + 1$.
end for
Convert the reactant molecular graph to a SMILES string.

Table 1. Distribution of 10 recognized reaction types

Reaction type	Reaction type name	# Examples
1	Heteroatom alkylation and arylation	15151
2	Acylation and related processes	11896
3	C-C bond formation	5662
4	Heterocycle formation	909
5	Protections	672
6	Deprotections	8237
7	Reductions	4614
8	Oxidations	811
9	Functional group interconversion (FGI)	1834
10	Functional group addition (FGA)	230

B.2. Atom and Bond Features

We adopt the atom and bond features design from Yan et al. (2020), with all feature computations carried out using the rdkit library. The atom features employed in MARS are summarized in Table 2. These features are primarily represented using one-hot encoding, except for the atomic mass, which is a real number scaled to a consistent order of magnitude.

Table 3 outlines the bond features employed in MARS. These features are also represented using one-hot encoding.

B.3. Notation

We provide a comprehensive list of symbols used throughout the paper to facilitate a better understanding of the presented concepts and equations, as shown in Table 4.

Table 2. Atom Feature used in MARS. All features are one-hot encoding, except the atomic mass is a real number scaled to be on the same order of magnitude.

Feature	Description	Size
Atom type	Type of atom (i.e. C, N, O), by atomic number	17
# Bond	Number of bonds the atom is involved in	7
Formal charge	Interger electronic charge assigned to atom	5
Chirality	Unsoecified, tetrahedral CW/CCW, or other	4
# Hs	Number of bonded Hydrogen atom	5
Hybridization	sp, sp2, sp3, sp3d, or sp3d2	5
Aromaticity	Whether this atom is part of an aromatic system	1
Atomic mass	Mass of the atom, divided by 100	1
Reaction type	The specified reaction type if it exists	10

Table 3. Bond Feature used in MARS.

Feature	Description	Size
Bond type	Single, double, triple, or aromatic.	4
Conjugation	Whether the bond is conjugated.	1
In ring	Whether the bond is part of a ring.	1
Stereo	None, any, E/Z or cis/trans.	6

Table 4. Notation

Notation	Short Explanation
G_*	Molecular graph
$\mathcal{V}$	Set of atoms
$\mathcal{E}$	Set of bonds
Z	Motif vocabulary and motif
a_j	Attachment atom j
q_j	Interface-atom j
π_t	Graph editing action at t step
o_t	Edit object at t step
τ_t	Edit state at t step
s_i	Editing score of edit object i
u_t	The output vector of GRU at t step
ψ_t	The vector derived from molecular graph embedding and the output of GRU at t step
$\sigma_*(\cdot)$	Linear layer of neural networks with any nonlinear activation
$f_*(\cdot)$	Linear layer of neural networks with any nonlinear activation, mapping entities to vectors
$\boldsymbol{h}_*$	Embedding vector

B.4. Baselines

We benchmark the performance of our proposed method against a comprehensive set of competitors, encompassing both template-based and template-free approaches. These competitors represent state-of-the-art solutions in the field of retrosynthesis prediction. The diversity of these methods offers a robust basis for evaluating the efficacy of our method.

For template-based models, we consider three prominent contenders:

- **RetroSim** (Coley et al., 2017) selects reaction centers based on Morgan fingerprint similarity between target molecules and known precedents.
- **NeuralSym** (Segler and Waller, 2017) combines a fully-connect layer and a deep highway network to learn knowledge of potential correlations between molecular functional groups and reactions.
- **GLN** (Dai et al., 2019) models the joint probability of single-step retrosynthesis to select templates and generate reactants.

Template-free models can be divided into five sequence-based models and five graph-based models. For sequence-based models, we consider five models:

- **SCROP** (Zheng et al., 2019) combines an extra Transformer to correct predicted SMILES strings.
- **RetroPrime** (Wang et al., 2021) uses two Transformers to model reaction center identification and synthons completion, respectively.
- **Retroformer** (Wan et al., 2022) jointly learns the sequential and graphical information of molecules using a Transformer-based local-global Encoder-Decoder model.

- **DualTF** (Sun et al., 2021) unifies sequence-based and graph-based models using energy functions and uses an extra order model to help inference.
- **Chemformer** (Irwin et al., 2022) is a BARt-based chemical language model with 230 million learnable weights pre-trained on 100 million molecules.

For graph-based models, we consider five models:

- **G2Gs** (Shi et al., 2020a) employs a graph neural network to select reaction centers and generates reactants using a variational autoencoder.
- **RetroXpert** (Yan et al., 2020) leverages a graph neural network to predict disconnections and regards reactant generation as a sequence translation task.
- **GraphRetro** (Somnath et al., 2021) determines the synthon through an edit prediction model and then performs a single full-connected network to complete the synthons by using predefined leaving groups.
- **MEGAN** (Sacha et al., 2021) defines five graph editing actions, using two stacked graph attention networks to perform retrosynthesis predictions.
- **G2GT** (Lin et al., 2023) utilizes a Graphormer to encode the product molecular diagram as a representation vector, and then uses a decoder to convert the representation vector into a graph sequence in an autoregressive way, which represents the graph structure of the reactants.

C. Parameter Analysis

In this section, we delve into the key factors that affect the performance of MARS. We systematically explore and analyze the effects of the various components that contribute to the effectiveness of the model. Specifically, we investigate the impact of different graph encoders, representation readout functions, dimension sizes, and the number of GTN layers, shedding light on the choices that optimize MARS's performance.

C.1. Effects of Graph Encoder

Graph encoders, which are responsible for learning representations of nodes by aggregating information from their neighbors, are used to capture topological information of molecular graphs. We investigate the effect of different graph encoders on the performance of MARS, including GCN (Kipf and Welling, 2016), GAT (Veličković et al., 2017), GraphSAGE (Hamilton et al., 2017) and GTN (Shi et al., 2020c). As shown in Fig. 1(a), GTN outperforms the other graph encoders. This is because the self-attention module in GTN can better fuse bond features into the atom representations, leading to more effective learning of the molecular graph.

C.2. Effects of Representation Readout Function

The representation readout function is utilized to aggregate all atom representations to obtain the molecular graph representation. We compare the effects of several readout functions on the performance of MARS, including max, sum, mean and attention pooling. Fig. 1(b) shows the Top-k accuracy of different readout functions. We find that attention pooling achieves the best performance for learning the representation of molecular graphs. This is because attention pooling can better extract information from atom representations that benefit downstream tasks.

C.3. Effects of Dimension Size

Embedding size K has a significant impact on performance. As shown in Figure 1(c), we test the performance of MARS when K takes on values of 64, 128, 256, 512, 1024. We find that the Top-1 accuracy is optimal when the embedding size is 512, and the performance does not increase further when the embedding size is 1024. For larger k, the accuracy of MARS is not sensitive to K. This demonstrates that 512 dimensions are sufficient for our model to perform optimally.

C.4. Effects of the Number of GTN Layers

We show the effects of the number of GTN layers in Figure 2. The findings demonstrate a gradual increase in the Top-1 and Top-3 accuracy of the model as the count of GTN layers is increased. At the same time, the performance of Top-K stabilizes after K exceeds 3. Remarkably, the model achieves its optimal Top-1 performance when the number of GTN layers is configured at 6. However, increasing the GTN layer to 7 induces a slight reduction in the Top-1 accuracy of the model due to overfitting. These observations highlight the efficacy of employing 6 GTN layers for superior representation learning of molecular graphs.

D. Visualization of Concatenating Multiple Motifs to Complete Synthons

We present three illustrative examples in Figure 3, each exemplifying the synthon completion through the amalgamation of multiple motifs. Notably, Example (a) represents a Top-2 prediction, demonstrating the accuracy and predictive capability of our model in predicting reactants. Also worth noting are the two remaining examples, where the leaving groups are formed by combining motifs absent from the training set. This not only highlights the model's ability to predict novel reactants, but also serves as a compelling testament to the inherent flexibility of our proposed motif-based approach.

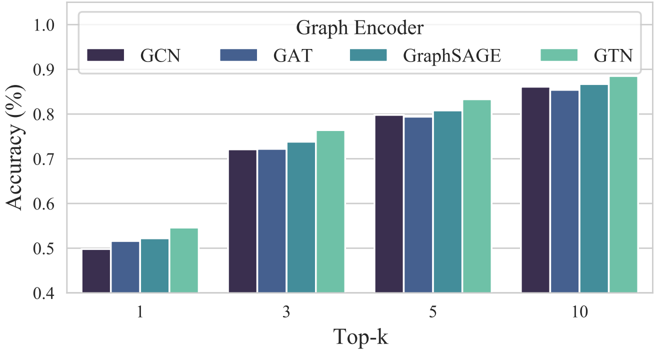

(a)

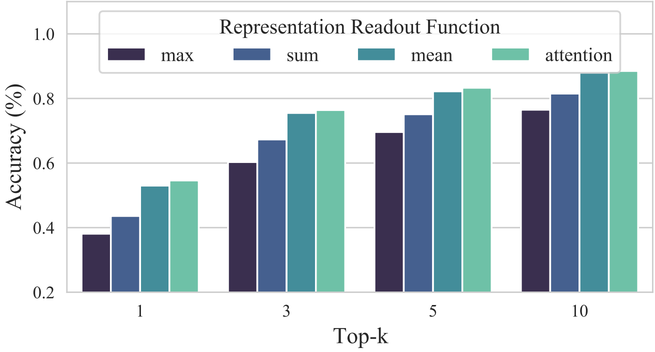

(b)

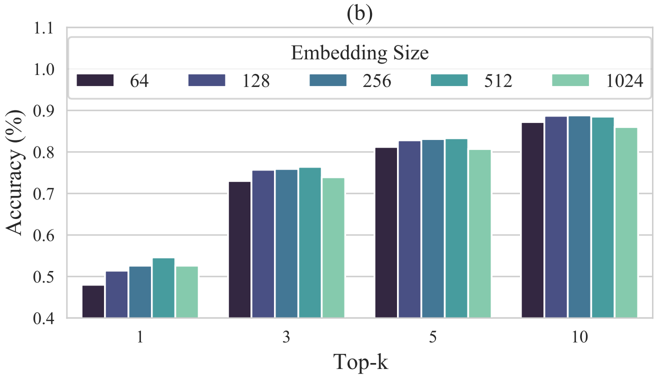

(c)

Fig. 1: (a). Performance comparison of different graph encoders. (b). Performance comparison of different representation readout functions. (c). Performance comparison of different dimension sizes.

E. Related works

E.1. Molecular Graph Generation Methods

The field of molecular graph generation has seen various approaches (Madhawa et al., 2019; Zang and Wang, 2020; Luo et al., 2021) aimed at generating chemically valid molecules with specific chemical properties. MolGAN (De Cao and Kipf, 2018) generates molecules via generative adversarial networks. JT-VAE (Jin et al., 2018) first decomposes a molecular graph into disconnected subgraphs and then designs a junction tree variational autoencoder for molecule generation. Recently, autoregressive-based models have gained much attention in molecular graph generation. GCPN (You et al., 2018) formulates molecular graph generation as a Markov Decision Process. MolecularRNN (Popova et al., 2019) utilizes a recurrent neural network to generate the nodes and edges. GraphAF (Shi et al., 2020b) designs a flow-based autoregressive model to dynamically generate nodes and edges based on historical subgraph structures. Our proposed method can be considered as a conditional molecular graph generation method based on an autoregressive model.

E.2. Template-free Retrosynthesis Prediction Methods

Template-free methods are data-driven methods that can be divided into sequence-based and graph-based methods. Sequence-based methods (Tetko et al., 2020; Wang et al., 2021; Mao et al., 2021) leverage natural language processing (NLP) techniques and treat the

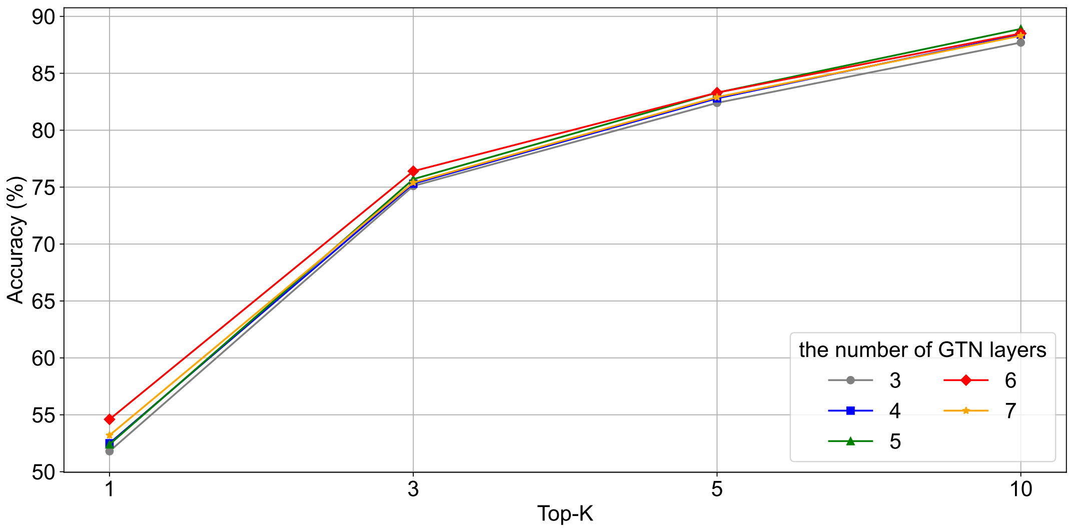

Fig. 2: Performance comparison of different numbers of Graph Transformer Network Layers.

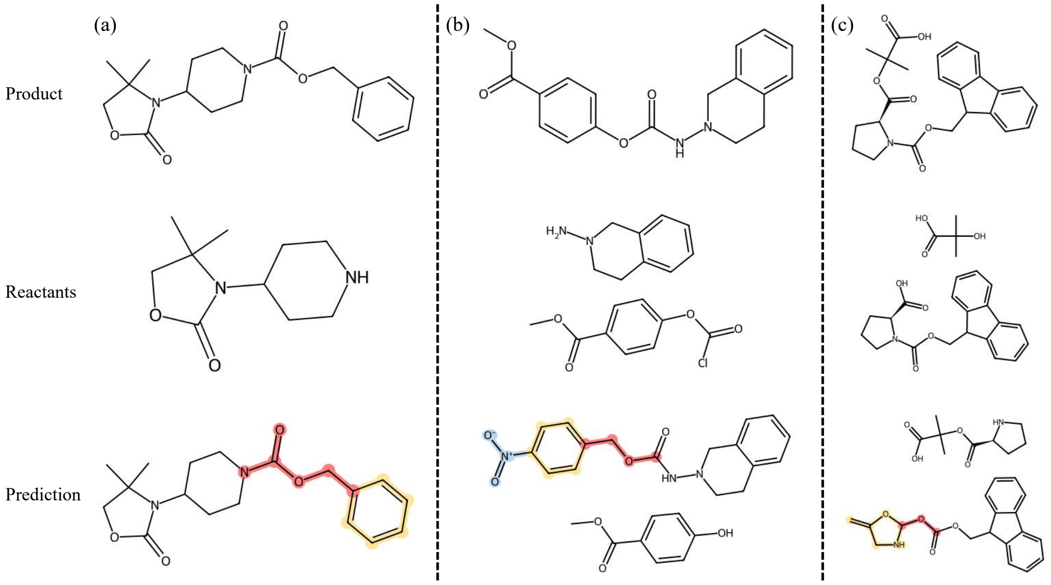

Fig. 3: Examples of complete synthons by concatenating motifs. Different colored substructures represent different motifs.

retrosynthesis task as a machine translation problem, with molecules represented as SMILES strings. For example, Seq2seq (Liu et al., 2017) and Transformer (Karpov et al., 2019) simply apply machine translation models to retrosynthesis tasks, resulting in the generation of ineffective molecules. To remedy the grammatically incorrect output in the previous models, SCROP adds a syntax corrector to correct the output smiles to achieve better performance than the vanilla Transformer. RetroPrime (Wang et al., 2021) uses two Transformers to translate products to synthons and synthons to reactants, respectively. Chemformer (Irwin et al., 2022) fine-tunes a chemical large language model to translate the product smiles into the reactant smiles. Although these methods simplify retrosynthetic models, most of them ignore the rich structural information in molecular graphs and are poorly interpretable. To solve this problem, Retroformer (Wan et al., 2022) incorporates graphical information to predict the reaction centers and then translates synthons into reactants.

Graph-based methods (Shi et al., 2020a; Sacha et al., 2021; Han et al., 2022), on the other hand, model the retrosynthesis task as two steps: i) break the target molecule into incomplete molecules called synthons, and then ii) complete them into reactants using subgraph units such as atoms or leaving groups. For instance, methods such as G2Gs (Shi et al., 2020a), RetroXpert (Yan et al., 2020), and GraphRetro (Somnath et al., 2021) build two independent models to implement the above steps, respectively. MEGAN (Sacha

et al., 2021) constructs an end-to-end graph generative model while completing synthon with individual atoms and benzene. Our work is closely related to graph-based models but fundamentally different from the above methods. First, rather than treating reaction center identification and synthon completion as two completely independent subtasks like (Shi et al., 2020a; Yan et al., 2020; Somnath et al., 2021), our work integrates these two subtasks into an end-to-end framework. Second, compared to the high prediction complexity of completing synthons with small units (Sacha et al., 2021), adding motifs to synthons can greatly reduce the length of the prediction sequence. It is worth noting that motifs are distinct from the leaving groups proposed by (Somnath et al., 2021) and the differences are discussed in subsection 3.2.

References

C. W. Coley, L. Rogers, W. H. Green, and K. F. Jensen. Computer-assisted retrosynthesis based on molecular similarity. *ACS central science*, 3(12):1237–1245, 2017.

H. Dai, C. Li, C. Coley, B. Dai, and L. Song. Retrosynthesis prediction with conditional graph logic network. *Advances in Neural Information Processing Systems*, 32:8872–8882, 2019.

N. De Cao and T. Kipf. Molgan: An implicit generative model for small molecular graphs. *arXiv preprint arXiv:1805.11973*, 2018.

M. Fey and J. E. Lenssen. Fast graph representation learning with pytorch geometric. *arXiv preprint arXiv:1903.02428*, 2019.

W. Hamilton, Z. Ying, and J. Leskovec. Inductive representation learning on large graphs. *Advances in neural information processing systems*, 30, 2017.

P. Han, P. Zhao, C. Lu, J. Huang, J. Wu, S. Shang, B. Yao, and X. Zhang. Gnn-retro: Retrosynthetic planning with graph neural networks. In *Proceedings of the AAAI Conference on Artificial Intelligence*, volume 36, pages 4014–4021, 2022.

R. Irwin, S. Dimitriadis, J. He, and E. J. Bjerrum. Chemformer: a pre-trained transformer for computational chemistry. *Machine Learning: Science and Technology*, 3(1):015022, 2022.

W. Jin, R. Barzilay, and T. Jaakkola. Junction tree variational autoencoder for molecular graph generation. In *International conference on machine learning*, pages 2323–2332. PMLR, 2018.

P. Karpov, G. Godin, and I. V. Tetko. A transformer model for retrosynthesis. In *International Conference on Artificial Neural Networks*, pages 817–830. Springer, 2019.

T. N. Kipf and M. Welling. Semi-supervised classification with graph convolutional networks. *arXiv preprint arXiv:1609.02907*, 2016.

Y. Li, D. Tarlow, M. Brockschmidt, and R. Zemel. Gated graph sequence neural networks. *arXiv preprint arXiv:1511.05493*, 2015.

Y. Li, C. Gu, T. Dullien, O. Vinyals, and P. Kohli. Graph matching networks for learning the similarity of graph structured objects. In *International conference on machine learning*, pages 3835–3845. PMLR, 2019.

Z. Lin, S. Yin, L. Shi, W. Zhou, and Y. J. Zhang. G2gt: Retrosynthesis prediction with graph-to-graph attention neural network and self-training. *Journal of Chemical Information and Modeling*, 63(7):1894–1905, 2023.

B. Liu, B. Ramsundar, P. Kawthekar, J. Shi, J. Gomes, Q. Luu Nguyen, S. Ho, J. Sloane, P. Wender, and V. Pande. Retrosynthetic reaction prediction using neural sequence-to-sequence models. *ACS central science*, 3(10):1103–1113, 2017.

Y. Luo, K. Yan, and S. Ji. Graphdf: A discrete flow model for molecular graph generation. In *International Conference on Machine Learning*, pages 7192–7203. PMLR, 2021.

K. Madhawa, K. Ishiguro, K. Nakago, and M. Abe. Graphnvp: An invertible flow model for generating molecular graphs. *arXiv preprint arXiv:1905.11600*, 2019.

K. Mao, X. Xiao, T. Xu, Y. Rong, J. Huang, and P. Zhao. Molecular graph enhanced transformer for retrosynthesis prediction. *Neurocomputing*, 457:193–202, 2021.

A. Paszke, S. Gross, F. Massa, A. Lerer, J. Bradbury, G. Chanan, T. Killeen, Z. Lin, N. Gimelshein, L. Antiga, et al. Pytorch: An imperative style, high-performance deep learning library. *Advances in neural information processing systems*, 32, 2019.

M. Popova, M. Shvets, J. Oliva, and O. Isayev. Molecularrnn: Generating realistic molecular graphs with optimized properties. *arXiv preprint arXiv:1905.13372*, 2019.

M. Sacha, M. Błaz, P. Byrski, P. Dabrowski-Tumanski, M. Chrominski, R. Loska, P. Włodarczyk-Pruszynski, and S. Jastrzebski. Molecule edit graph attention network: modeling chemical reactions as sequences of graph edits. *Journal of Chemical Information and Modeling*, 61(7):3273–3284, 2021.

M. H. Segler and M. P. Waller. Neural-symbolic machine learning for retrosynthesis and reaction prediction. *Chemistry–A European Journal*, 23(25):5966–5971, 2017.

C. Shi, M. Xu, H. Guo, M. Zhang, and J. Tang. A graph to graphs framework for retrosynthesis prediction. In *International Conference on Machine Learning*, pages 8818–8827. PMLR, 2020a.

C. Shi, M. Xu, Z. Zhu, W. Zhang, M. Zhang, and J. Tang. Graphaf: a flow-based autoregressive model for molecular graph generation. *arXiv preprint arXiv:2001.09382*, 2020b.

Y. Shi, Z. Huang, S. Feng, H. Zhong, W. Wang, and Y. Sun. Masked label prediction: Unified message passing model for semi-supervised classification. *arXiv preprint arXiv:2009.03509*, 2020c.

V. R. Somnath, C. Bunne, C. Coley, A. Krause, and R. Barzilay. Learning graph models for retrosynthesis prediction. *Advances in Neural Information Processing Systems*, 34, 2021.

R. Sun, H. Dai, L. Li, S. Kearnes, and B. Dai. Towards understanding retrosynthesis by energy-based models. *Advances in Neural Information Processing Systems*, 34, 2021.

I. V. Tetko, P. Karpov, R. Van Deursen, and G. Godin. State-of-the-art augmented nlp transformer models for direct and single-step retrosynthesis. *Nature communications*, 11(1):5575, 2020.

A. Vaswani, N. Shazeer, N. Parmar, J. Uszkoreit, L. Jones, A. N. Gomez, Ł. Kaiser, and I. Polosukhin. Attention is all you need. In *Advances in neural information processing systems*, pages 5998–6008, 2017.

P. Veličković, G. Cucurull, A. Casanova, A. Romero, P. Lio, and Y. Bengio. Graph attention networks. *arXiv preprint arXiv:1710.10903*, 2017.

Y. Wan, C.-Y. Hsieh, B. Liao, and S. Zhang. Retroformer: Pushing the limits of end-to-end retrosynthesis transformer. In *International Conference on Machine Learning*, pages 22475–22490. PMLR, 2022.

X. Wang, Y. Li, J. Qiu, G. Chen, H. Liu, B. Liao, C.-Y. Hsieh, and X. Yao. Retroprime: A diverse, plausible and transformer-based method for single-step retrosynthesis predictions. *Chemical Engineering Journal*, 420:129845, 2021.

C. Yan, Q. Ding, P. Zhao, S. Zheng, J. YANG, Y. Yu, and J. Huang. Retroxpert: Decompose retrosynthesis prediction like a chemist. In H. Larochelle, M. Ranzato, R. Hadsell, M. F. Balcan, and H. Lin, editors, *Advances in Neural Information Processing Systems*, volume 33, pages 11248–11258. Curran Associates, Inc., 2020.

J. You, B. Liu, Z. Ying, V. Pande, and J. Leskovec. Graph convolutional policy network for goal-directed molecular graph generation. *Advances in neural information processing systems*, 31, 2018.

C. Zang and F. Wang. Moflow: an invertible flow model for generating molecular graphs. In *Proceedings of the 26th ACM SIGKDD International Conference on Knowledge Discovery & Data Mining*, pages 617–626, 2020.

S. Zheng, J. Rao, Z. Zhang, J. Xu, and Y. Yang. Predicting retrosynthetic reactions using self-corrected transformer neural networks. *Journal of Chemical Information and Modeling*, 60(1):47–55, 2019.
